# A Low-Cost Silica Fiber/Epoxy Composite with Excellent Dielectric Properties, and Good Mechanical and Thermal Stability

**DOI:** 10.3390/ma16237410

**Published:** 2023-11-29

**Authors:** Imran Haider, Iftikhar Hussain Gul, Malik Adeel Umer, Mutawara Mahmood Baig

**Affiliations:** Thermal Transport Laboratory, Department of Materials Engineering, School of Chemical & Materials Engineering (SCME), National University of Sciences & Technology (NUST), Islamabad 44000, Pakistan

**Keywords:** reinforced composites, low dielectric, microwave properties, tensile properties

## Abstract

In many electronic applications, the dielectric and structural properties of reinforced composites are vital. In this research work, the influence of fiber proportion on the properties of a silica fiber/epoxy (SFE) composite was investigated. The structure, morphology, dielectric constant and loss factor, mechanical properties, and thermal stability were determined. The increase of wt.% of silica fiber (SiO_2 (f)_) x = 30 to 90, reduced the dielectric constant (εr) and dielectric loss (δ) of the SFE composite from their original values to 18.9% and 48.5%, lowering local charge displacement towards the applied electric field. The SFE composite showed higher mechanical properties with the increase in SiO_2 (f)_, x = 30 to 80, the tensile strength (UTS) was raised from 91.6 MPa to 155.7 MPa, the compression strength (UCS) was increased from 261.1 MPa to 409.6 MPa and the flexural strength was enhanced from 192.3 MPa to 311.9 MPa. Upon further addition of SiO_2 (f)_ to the composite, i.e., x = 90, the mechanical properties were reduced a little, but the dielectric properties were not changed. Increasing SiO_2 (f)_ improved the thermal stability as weight loss was found to be 69% (x = 30) and 24% (x = 90), and average moisture absorption was found to be 1.1 to 1.8%. A silica fiber/epoxy composite, for microelectronics, can be made from a low-cost fiber, and its dielectric properties as well as its mechanical and thermal stability can be tuned or improved by varying fiber fractions.

## 1. Introduction

In interconnections, printed circuit boards, and airplane skin materials, composite material dielectric properties are important for their electrical transparency or microwave reflection [1]. Silica fiber-reinforced ceramic matrix composites are wave-transparent materials owing to their excellent dielectric properties, good ablation resistance, fine thermal shock damage resistance, and chemical stability [2,3,4]. Fiber-reinforced composites (FRC) have high specific strength and stiffness, high temperature, and fatigue resistance [5,6]. The mechanical performance of FRC is influenced by the phases and their interactions [6,7,8]. For polymer composites, the dielectric properties are based on component volume fractions [9,10]. Hybrid composites are often designed from multiple reinforcements to attain preferred electric and mechanical properties [11,12,13]. Silica fibers (SiO_2_ > 99.95%) have many superior properties at high temperatures such as a low dielectric constant, small coefficient of thermal expansion, low thermal conductivity, and mechanical strength [14].

Polymer-based composites are limited due to their relatively low working temperatures and dielectric instability, which means that they fail to meet increasing demands of industrial applications. Therefore, it is urgent to fabricate polymer-based composites with stable dielectric properties over a wide range of temperature and frequency [15].

Cyanate ester (CE) has excellent dielectric properties, adhesion, mechanical strength, thermal stability, and very low moisture absorption [16,17]. Electromagnetic wave transparent materials, called “electronic windows”, are used on high-speed vehicles and aircraft antenna windows for harsh environments, and must possess excellent dielectric, mechanical, and thermal stability [18,19,20,21]. Glycidyl polyhedral oligomer silsesquioxane (G-POSS) and γ-aminopropyltriethoxy silane coupling agent (KH-550) were introduced into SF-reinforced Cyanate ester (CE) resin composites to exhibit comprehensive performance, showing great potential for dielectric applications [22].

Production cost, quality, and shape requirements are important [23] when selecting a composite manufacturing process, such as compression molding, hand lay-up, spray-up, vacuum bagging, resin transfer molding, autoclave molding, filament winding, pultrusion, injection molding, prepregging, and stamp forming [24]. For the glass fiber–epoxy mixture, its effective permittivity is a function of frequency, fiber volume fraction, cure state, porosity, and fiber orientation, and the empirical mixing formula can be adopted for the prediction of the effective dielectric constant, such as Wiener limits, Maxwell Garnett formula, and the Looyenga formula [25], as it mainly depends on the dielectric properties of the fiber and resin [26]. To achieve high performance, the effect of fiber content on the tensile properties of reinforced composites is of interest and significance for many researchers [27] as the reinforcement raises the composite strength and modulus [28]. Glass fibers are the earliest and most widely used reinforcement in radomes due to their low cost, low ε, δ, excellent insulation and mechanical properties [29].

Reinforced composites from D-glass (SiO_2_~72–75%) and quartz fibers (SiO_2_ ≥ 99.9) [14] are used in electromagnetic wave transparent applications. Along with the highest cost, Quartz fibers possess outstanding thermal and mechanical properties with very low and stable dielectric loss [30]. D-glass fibers are specially developed for high-performance radomes applications but at the expense of mechanical properties [31]. High-silica-glass fabric is made from the lowest cost variant and the weakest silica fibers (SiO_2_ = 95–99.5%) by acid leaching of A-or E-glass fabric. It is commonly being used in fireproofing and industrial insulation applications, has found limited use in aircraft and aerospace applications [32]. High-silica-glass fabric is desirable and cost-effective heat-insulating materials for less demanding uses than those which require pure or ultrapure silica fibers [33,34].

For various electronic applications, a huge number of dielectric materials have been developed but a single material cannot conform to all requirements along with minimum cost. The basic characteristic property of a very good low dielectric material is that it possesses a very low dielectric constant (ε ≤ 4.5) and dielectric loss (δ ≤ 0.06), however, low cost, good mechanical properties, and environmental resistance are important as well [35].

E-GFs (SiO_2_~54%) possess excellent mechanical strength and a medium cost but higher ε (5.4~6), S-GFs (SiO_2_~64–66%) have high strength, ε (5.1), elasticity, and cost. D-GFs have the lowest ε and δ among all GFs and lower mechanical properties with higher cost, Quartz GFs possess low ε and δ close to D-GFs, have the best impact, chemical and temperature resistance, but also have the highest cost [36].

Phenolic, polyester, epoxy, cyanate ester, and bismaleimide resins are used in making composites for various electronic and aerospace applications [36]. Polyester resin is general purpose and has lower strength, phenolic resin is a good choice due to its high strength but needs special handling, storage, curing time, and multiple cycles, cyanate ester is the best for such applications but has the highest cost, bismaleimide resin is high-temperature-resistant and high-cost with matrix embrittle issues. In comparison with these, epoxy resin offers very good mechanical properties, easy handling, and processing, along with low cost [35,36,37].

Many researchers have studied the physical, mechanical, electrical, and thermal properties of glass-fiber-reinforced composites and found that fiber–matrix nature, proportion, orientation, direction, quantity, fiber–matrix adhesion, salinization, cyclic temperature changes, long-term exposure conditions, hydrolytic degradation of the polymer, and fillers all contribute towards the composite’s performance. Each of the contributing parameters has its significance in specified application, long-term strength, stability, durability, and performance [35,36,37,38,39].

Long-term stability is an important feature of composite materials, and it depends on multiple variables, i.e., temperature, humidity, time, UV light, dust, fatigue, wind, weather, chemical and mechanical factors, inside physical cracking, or a combination of these [40,41,42]. The impacts of long-term use and their significance were not studied in this work.

This work is aimed at fabricating a low-cost dielectric composite possessing good mechanical and thermal stability. A commercial grade fabric named “High-Silica glass” has similar dielectric properties to the high-cost reinforcements. Here, in this work, a commercial Di-glycidyl Ether of Bisphenol-A(DGEBA) epoxy resin was used due to its good mechanical and electrical characteristics [16] and low cost compared to other thermoset resins.

## 2. Materials and Methods

### 2.1. Materials

Raw materials used in manufacturing low-dielectric composites [29] are compared in Table 1 and Table 2. “E” refers to epoxy resin, “SF” to silica fiber, and subscript (f) to the fabric.

SFE composites were prepared from a low-cost commercial liquid DGEBA resin, Magic^®^ (Epoxy Industries Pakistan, Karachi, Pakistan), density 1.90–1.95 g/cc, viscosity 10,300 cPs, and epoxy content 23%. Commercial-grade silica fabric (BWT260-82) TESCNICA, fiber dia. ~8.0 to 10.0 μm, density ~2.35 g/cc, ply thickness = 0.25 mm, softening point ~1600 °C, was used.

### 2.2. Fabrication of Composite

**Figure 1:**
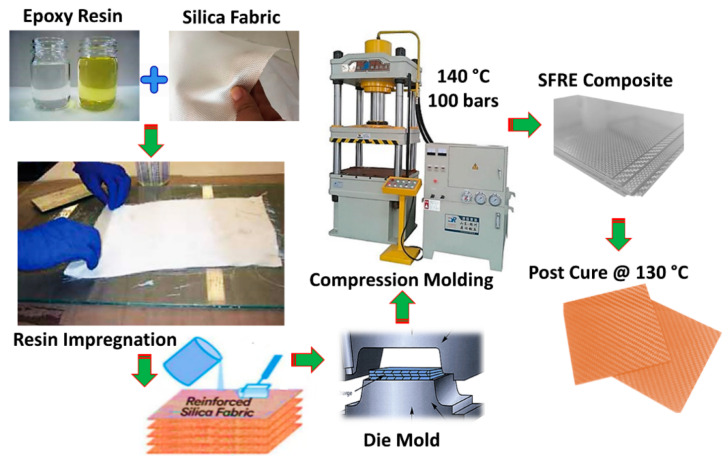
Schematic (SFE composite fabrication).

Unidirectional woven silica fabric was cut into layers of (200 × 200), washed with ethanol and dried. Varying weight proportions of silica fabric (SF) to epoxy (E) (x = 30%, 40%, 50%, 60%, 70%, 80%, and 90%) were fabricated as shown in Figure 1. The scheme included: pre-treatment of silica fabric, preparation of epoxy matrix, soaking of fabric sheets in a resin matrix, layup, compression, and curing. Experimental conditions are mentioned in Table 3 and identification scheme of SFE composites is presented in Table 4.

### 2.3. Characterization

SFE composite specimens were characterized for dielectric constant, loss tangent (PNA Network 8362B Agilent, Santa Clara, CA, USA), density (ASTM D-792) using analytical weighing balance (SHIMADZU-UW 3400 g), structure (FTIR—Perkin Elmer Spectrum, USA, XRD-STOE-Seifert X’Pert PRO), morphology by Scanning Electron Microscopy (JSM-6490A, EOL Japan), tensile strength (ASTM D 638), compression strength (ASTM D 6641), three-point bending strength (ASTM D-7264) by universal testing machine AGX-Plus (SHIMADZU Japan), weight loss (TGA Q600 SDT, TA instruments), and moisture absorption (ASTM-D 570) in both fiber directions using analytical weighing balance (SHIMADZU-UW 3400 g).

## 3. Results and Discussion

### 3.1. Density

**Figure 2:**
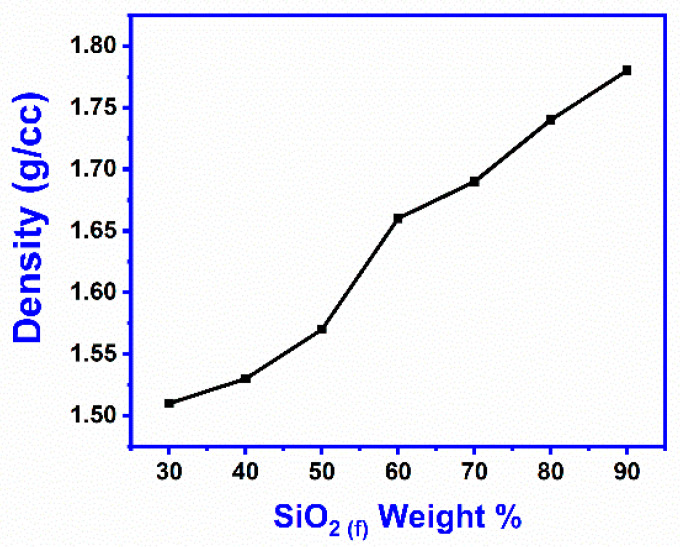
Density of SFE composites.

Figure 2 shows the density of SFE composites which ranged from 1.5 g/c to 1.78 g/cc. The density increased with increasing silica fiber levels in the composite, i.e., SiO2 (f) (x = 30 to 90). Low density (a quality of SFE composites) is a desirable property of fiber-reinforced composites, i.e., compactness, increased with the addition of SiO_2 (f)_ up to x = 70%, but by further adding silica fiber (i.e., x = 80 and x = 90) the density was further raised by 2%, which was said to be insignificant. It was found that a good fiber–matrix adhesion was achieved up to x = 70% by increasing the density to its maximum value.

### 3.2. FTIR

**Figure 3:**
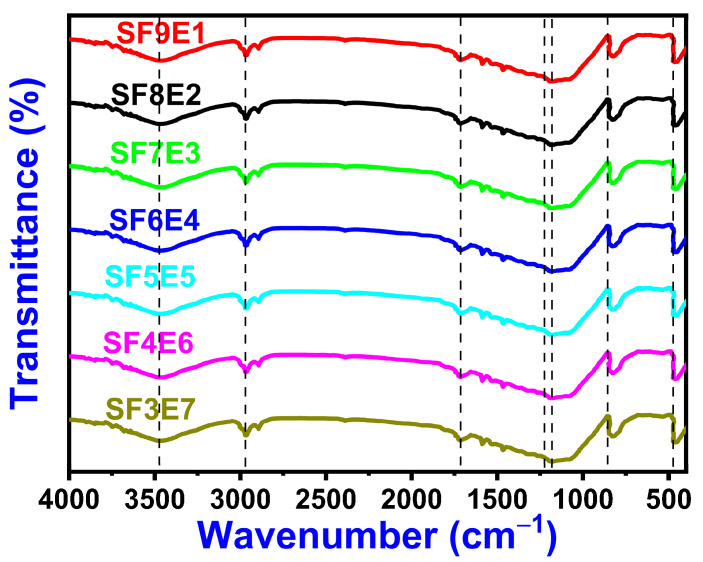
FTIR of SFE composites (30–90 wt.% of SiO_2 (f)_).

The presence of organic and inorganic networks in SFE composites was identified by the FTIR spectrum as shown in Figure 3. In these all-composite proportions, SiO_2 (f)_ (x = 30 to 90) exhibited similar bands at 3441.44 cm^−1^ and 2924 cm^−1^, OH stretching (hydroxyl linkage) [36] and the peak at 1643 cm^−1^ corresponded to the C = O double bond (polyamide) in the epoxy linkage. The clear Si-O-Si deformation band was observed at 468 cm^−1^ [36]. The absorption peak at 1177.9 cm^−1^ was attributed to the C-N-C stretching of tertiary amine to form an epoxy–ammine network. The bending peaks located at 1097 cm^−1^ were attributed to the symmetric and asymmetric stretching of C-O-C. The band observed at 800 cm^−1^ was due to aromatic C-H linkage deformation and was attributed as a possibility of the vibration modes of SiO_4_ tetrahedron.

### 3.3. X-ray Diffraction (XRD)

**Figure 4:**
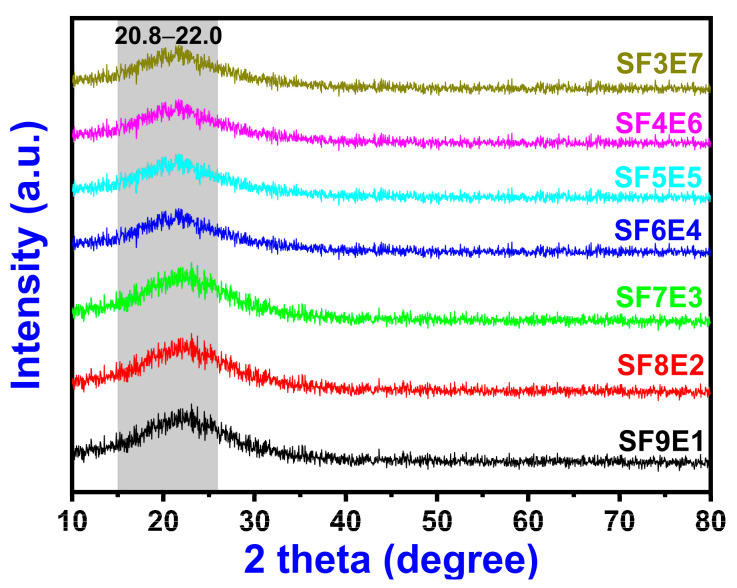
XRD patterns of SFE composites (30–90 wt.% of SiO_2 (f)_).

X-ray diffraction demonstrated the presence of (SiO_2_) glass fiber in epoxy resin, as in Figure 4, with an unidentifiable XRD peak. The Bragg’s angle 2θ ranged from 20.8–22.0° which confirmed the amorphous nature of SF3E7, SF4E6, SF5E5, SF6E4, SF7E3, SF8E2, and SF9E1 composites. The dispersion and adhesion of SiO_2 (f)_ and curing of the thermoset resin formed an amorphous phase without any additional peaks. The XRD patterns of the fabricated composites were typical X-ray diffractogram patterns of silica wave transparent composites [43,44], where there was no chance of crystallization of the fiber matrix as silica fabric is thermally stable.

### 3.4. Morphology

**Figure 5:**
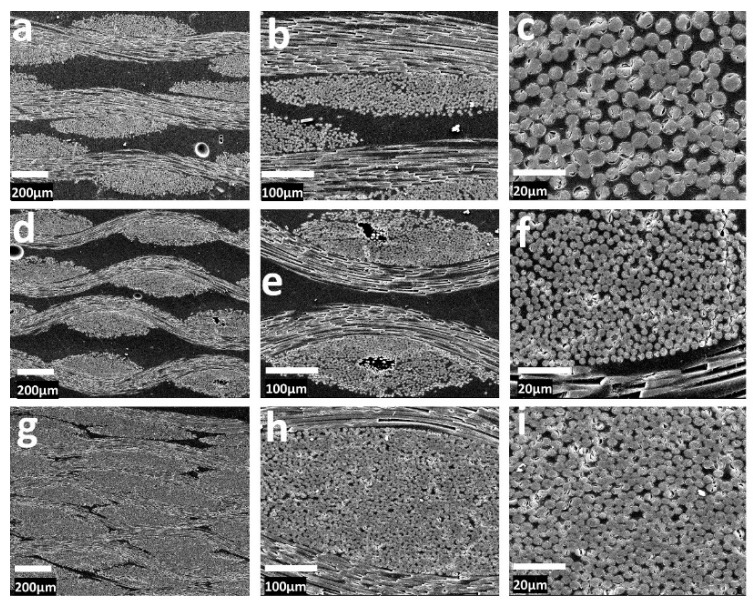
SEM of SFE composites, SF6E4 with 60% SiO_2 (f)_ [longitudinal (**a**,**b**) cross-sectional (**c**)], SF7E3 with SiO_2 (f)_ 70% [longitudinal (**d**,**e**) cross-sectional (**f**)] and, SF8E2 with SiO_2 (f)_ 70% [longitudinal (**g**,**h**) cross-sectional (**i**)].

The morphology of the SFE composites (SF6E4, SF7E3, and SF8E2) was observed by SEM, as shown in Figure 5, where adhesion of the matrix with reinforcing fibers can be seen SF6E4, Figure 5a–c. Minor free spaces were observed in the packed composite attributed to the air entrapped air during soaking, compaction, and early cross-linking of the epoxy matrix. Figure 5d–f are the SEM images of composite SF7E3, where SiO_2 (f)_ (x = 70), showed a better fiber–matrix adhesion than SF6E4. The appearance was similar to SF6E4 with a small number of fibers seeming to have been pulled out in some of the areas. There were fewer interfacial separations between fiber–matrix interfaces and small voids despite of higher fraction of reinforcement. Free spaces due to fiber slippage during the compaction of fabric sheets were present.

This localized discontinuity and uneven structural morphology are evident in Figure 5f. The SEM images of SF8E2, where x = 80, are shown in Figure 5g–i. Due to the higher SiO_2 (f)_ content, a more uniform distribution of fibers was created as compared to the SF6E4 and SF7E3 composites. Figure 5i showed the interfacial bonding between fiber–matrix interfaces where silica fiber plies were tightly packed and well-aligned due to higher SiO_2 (f)_% which reduced the fiber pull-out. Figure 5h,i showed a uniform and good fiber adhesion among all of these composites. Fiber–matrix adhesion and interfacial separations were compared to the loaded silica fibers [43,45].

**Figure 6:**
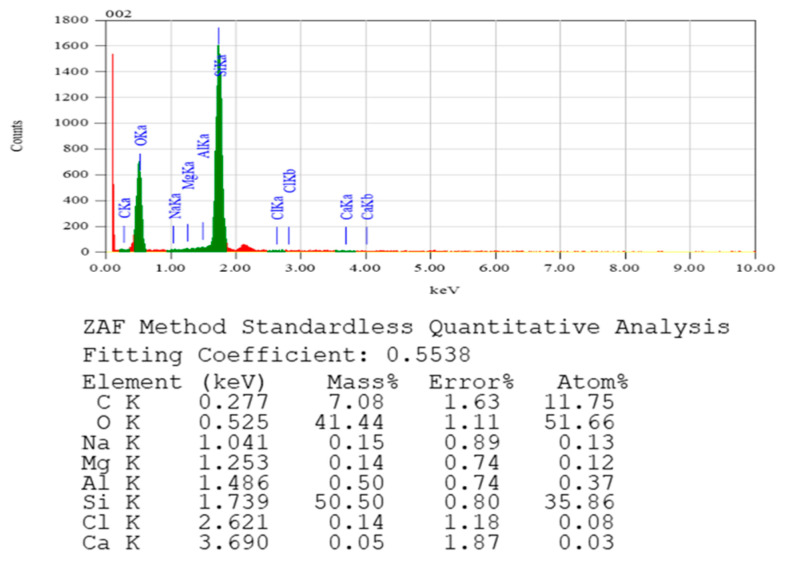
EDS of SFE composites.

Figure 6 showed the EDS spectra of SFE composite (SF8E2). FTIR, XRD, and SEM images represented a similar physical nature, while the elemental composition extracted from EDS revealed the amorphous nature of SFE composite. Minute proportions of sodium, magnesium, aluminum, and calcium were present as these were contained in the high-silica fibers. The presented EDS spectra were found to be in good agreement with the previously reported results and, generally, this qualitative information is considered as supporting evidence along with other characterizations [42].

### 3.5. Dielectric Constant

**Figure 7:**
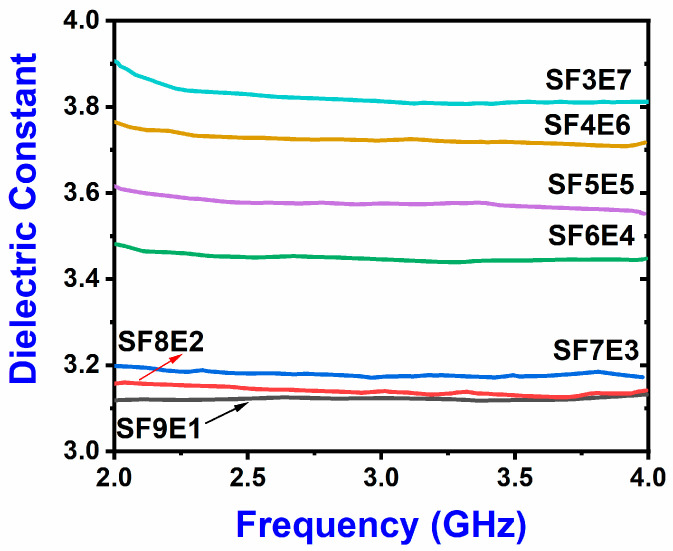
Dielectric constant of SFE composite.

In the fabricated SFE composites, the dielectric constant (ε_r_) as a function of frequency (in S-band) is shown in Figure 7. With the increase in SiO_2 (f)_ at x = 30, 40, 50, 60, 70, and 80%, the dielectric constant (εr) at 4GHz was decreased from 3.82, 3.73, 3.57, 3.45, and 3.19 to 3.17, respectively. However, the dielectric constant was unchanged even after further adding SiO_2 (f),_ up to x = 90. Comparatively, higher dielectric constants were observed in the SFE composite with SiO_2 (f)_ x = 30% to 60% than those where SiO_2 (f)_ x = 70%, 80%, and 90%. The constituent (SiO_2_ (f) and epoxy resin) formed the heterogeneous dielectric structure in which the increased wt.% of SiO_2 (f)_ created space charge polarization with the packed interfacial boundaries. The increased (x) wt.% of SiO_2 (f)_ decreased the hopping electrons and local charge displacement towards the electric field in low electric polarization. The dielectric dispersion was increased in the real and imaginary parts of the dielectric constants and thus the dielectric constants were decreased.

In SF7E3, SF8E2, and SF9E1, the charge migration and re-orientation of relatively polarizable molecules were restricted. Due to the curing (epoxy), the movement of free ions was stopped, which decreased the ionic conductivity. The fast growth of large epoxy networks reached a point where the contribution from dipole relaxation was dominant over the ionic conductivity. These all contributed to low dielectric constant values.

### 3.6. Dilecetric Loss Tangent

**Figure 8:**
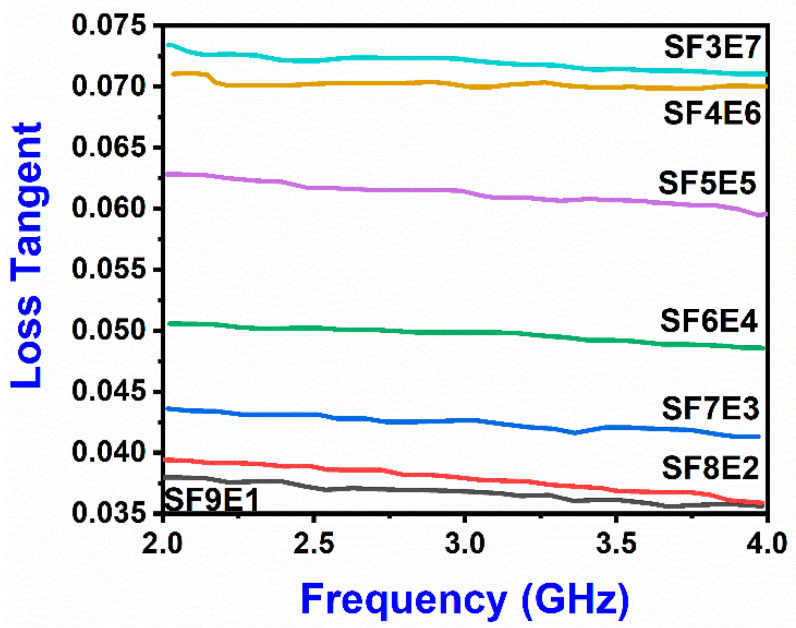
Dielectric tan loss of SFE composite.

The electromagnetic behavior of dielectric material is primarily described by its relative dielectric constant and tan (δ) loss. When interacting with the electromagnetic fields, the absorption of electromagnetic waves (within a dielectric material) is due to a high loss tangent (δ) which slows down the transmission of signals. The δ (4GHz) was decreased as 0.071, 0.070, 0.057, 0.047, 0.041, 0.036 and 0.036 corresponds to the increased (SiO_2 (f)_ wt.%) at x = 30, 40, 50, 60, 70, and 80, respectively, as shown by Figure 8. Since the constituent materials (SiO_2 (f)_ and epoxy resin) are non-magnetic, the low dielectric loss was found in the resultant SFE composite. The tangent loss reached its lowest value of 0.036 for SF8E2 (x = 80) and S9E1 (x = 90) and a comparatively higher dielectric loss factor was observed for SF3E7, SF4E6, SF5E5 and SF6E4 than those which contained SiO_2 (f)_ at x = 70, 80, 90. The higher proportion of SiO_2 (f)_ in the composite restricted the local charge displacement towards the electric field. Due to this restriction on the charge migration, polarization was reduced, and tangent loss was decreased. This lowest value of dielectric tan (δ) loss was evidence that charge polarization had been fixed. The lower energy dissipation was evident from the significant reduction (49%) of dielectric loss. Other contributing factors toward the tangent loss value, such as cure conditions, post-curing, resin properties, and plies orientation, were not included in this study.

### 3.7. Electrical Conductivity

Dielectric material possesses high resistivity as there are no free electrons to carry current, thus the electrical conductivity of these materials is very low [42]. SFE composite is made from insulating materials (SiO_2_ fiber and Epoxy resin). Due to this insulating nature, the resultant composite has low electrical conductivity. High-silica fibers possess high resistivity of ≥1000 Ω.m [46,47] and electrical conductivity is inversely proportional to resistivity. The electrical conductivity is denoted by the following relation σ = ω. ε_0_ × ε′ tan δ, where

ω = frequency
ε_0_ = 8.85 × 10^−12^
tan δ = ε′′/ε′

The σ was found at 9.65933 × 10^−20^,9.29892 × 10^−20^, 7.24717 × 10^−20^, 5.77487 × 10^−20^, 4.65801 × 10^−20^, 4.06431 × 10^−20^, 4.06431 × 10^−20^, which corresponds to the increased SiO_2 (f)_ (wt.% at x = 30 to 90).

### 3.8. Tensile Properties

#### 3.8.1. Ultimate Tensile Strength (UTS)

**Figure 9:**
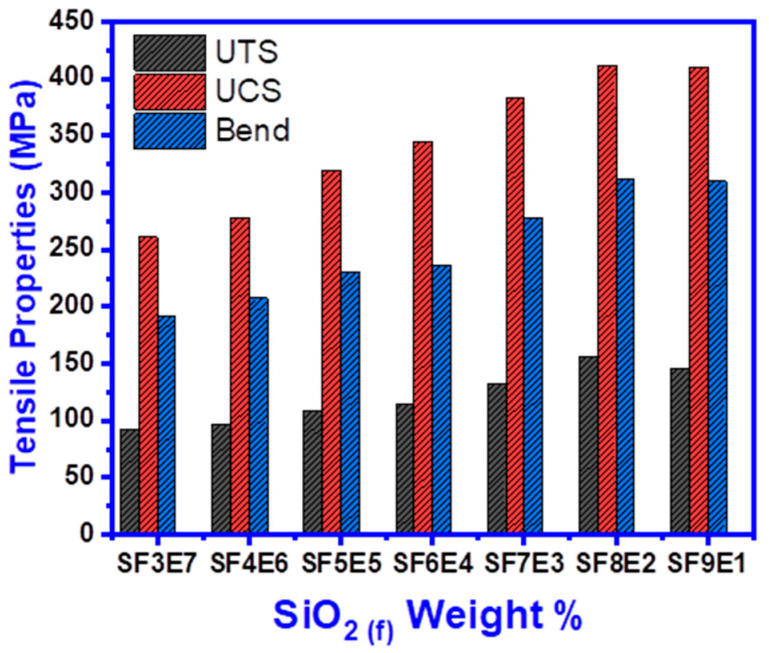
Tensile Properties of SFE composites (30−90 wt.% of SiO_2 (f)_).

The ultimate tensile strength (UTS) was increased from 91.6 to 155.7 MPa, corresponding to increased SiO_2 (f)_ proportion in the composite as shown in Figure 9. As the SiO_2 (f)_ (x) increased, UTS was increased from 91.6 MPa (at x = 30), 96.3 MPa (x = 40), 108.5 MPa (x = 50), 115.1 MPa (x = 60), 132.3 MPa (x = 70) and 155.7 MPa (x = 80), which was the highest value. The overall UTS was 41.1% increased, formed a tough composite structure, and resulted in failure at higher loads. Optimum fiber–matrix proportion is key to yielding the higher tensile strength of the composite. However, further addition of SiO_2 (f),_ x = 90, the tensile strength was reduced to 146.3 MPa which attributed that balance between fiber–thermoset resin was changed, and the adhesive bonding was reduced due to weak interfaces. Fibers in the composite were pulled at relatively lower loading due to higher fiber proportion.

#### 3.8.2. Ultimate Compression Strength (UCS)

Overall, the addition of the silica fibers enhanced the compressive strength (UCS), as shown in Figure 9, revealed that UCS increased from 261.06 MPa (at x = 30), 278.30 MPa (x = 40), 320.07 MPa (x = 50), 345.4 MPa (x = 60), 382.8 MPa (x = 70) and 409.64 MPa (x = 80) with the increase in SiO_2 (f)_ from 30 to 80 wt. %. From the initial value of UCS, it was 40.4% increased and reached to its highest value (409.64 MPa) for SF8E2 which revealed that SiO_2 (f)_ enabled it to bear high compressive loads. However, after approaching to maximum value, further addition of SiO_2 (f)_ resulted in a slightly lower UCS to 405.14 MPa (at x = 90). It seemed that stresses due to compressive load were borne by fibers, rather than the epoxy and fiber–matrix interface.

#### 3.8.3. Flexural Strength

Flexural strength represents the collective effects of tensile, compressive, and shear stresses liable for the failure of any material against the applied load. Upon increased wt.% of SiO_2 (f)_ from 30 to 80 wt.%, the flexural strength (Figure 9) was 41% increased. The flexural strength was 192.36 MPa (at x = 30), 207.05 MPa (x = 40), 230.02 MPa (x = 50), 235.75 MPa (x = 60), 277.2 MPa (x = 70) and the highest value reached 311.9 MPa (x = 80). With the onward addition (x = 90), the composite fractured at a 1% lower flexural load which showed that the flexural toughness of SF8E2 and SF9E1 were nearly similar. A similar value of flexural strength indicated that the toughness of the composite was transformed to become slightly brittle with the increasing fiber proportion.

### 3.9. Thermo-Gravimetric Analysis

**Figure 10:**
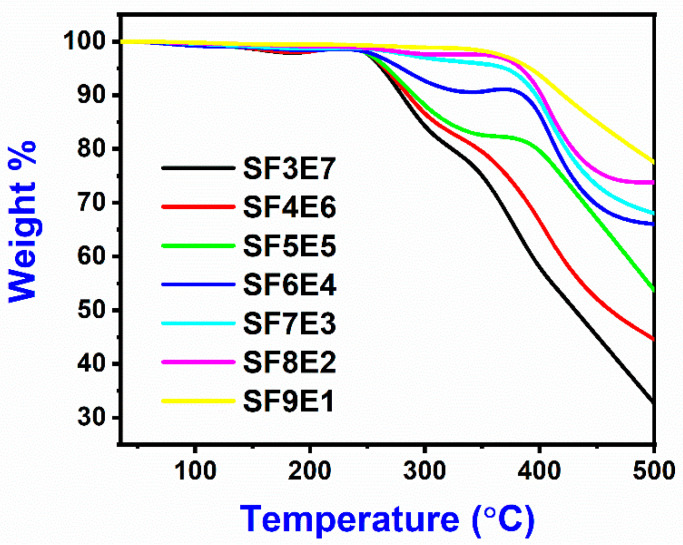
Thermogravimetric analysis of SFE composites.

Thermo-mechanical behavior of SFE composites against the rising temperature was determined by thermo-gravimetric analysis (TGA) as shown in Figure 10. The thermo-gram, showing the degradation profile by TGA curves, represented the major degradation which was due to the removal of the polymeric part from the composite. Cured thermoset resin and SiO_2 (f)_ both contributed to thermal resistance from 200 °C to 250 °C, where all the SFE composites samples remained stable. Above 250 °C, degradation started, and the major degradation was seen from 300 °C to 500 °C, with maximum weight loss. The de-binding in the multilayer fiber-reinforced composite was due to the softening of the organic part (polymeric resin) and the start of the degradation of epoxy. SFE composites with SiO_2 (f)_ at x = 60, 70, 80, and x = 90, offered a better thermal resistance due to higher SiO_2_ proportion. The degradation of the composite has two regions, from ambient to 250 °C, where these composites (SF3E7, SF4E6, SF5E5, SF6E4, SF7E3, SF8E2, and SF9E1) remained stable. From 300 °C to 500 °C the maximum degradation of SF3E7 was 68% (at x = 30). The polymeric part melted and then carbonized completely due to its inherent organic nature. However, in the second region, from 300 °C to 500 °C, the behavior of the samples at (x = 30, 40, 50) was found to be different from the samples at (x = 60, 70, 80, 90). It was very clear that the increased proportion of silica fibers (x = 60 to 90) formed a glassy substrate to protect the interface and thus reduced the degradation.

### 3.10. Moisture Absorption

Average moisture absorption of (SFE SF3E7, SF4E6, SF5E5, SF6E4, SF7E3, SF8E2, and SF9E1) was found to be 1.1 to 1.3% (longitudinal fiber direction) while in traverse fiber directions it was found to be 1.6 to 1.8%. Moisture absorption is significant in determining composite long-term strength and stability as it not only degrades the fiber–matrix bonding, but also mechanical properties (due to plasticizing effect), and electrical properties (due to high ε of water = 70). Moisture was absorbed in the exposed surface as moisture uptake is a function of the shape ratio (specimen surface area and volume) [40,42]. As described herein, in the traverse cut composites, 6.1% more moisture was absorbed than in longitudinal cut specimens due to the higher surface area and interfaces available for moisture absorption.

### 3.11. Property Comparison of Glass Fiber/Epoxy Composite

The properties of the glass fiber composite depend on multiple variables including the nature of the fiber, bonding matrix, their proportion, fiber orientation, direction, mixing ratios, processing methods, curing conditions (temperature, pressure, time, cycle, resin degassing, mixing, impregnation time, heating mode, heating rate, stacking sequence, pre-heating of raw materials and mold as well as post-curing. Besides many other contributing factors towards the resultant properties of the composite, to the best of our knowledge, the similar/related properties of glass fiber composites (D-GFs, E-GFs, Quart GFs, and high-silica glass fiber) are compiled in Table 5.

## 4. Conclusions

In this research work, the loading of SiO_2 (f)_ (w/w%) on properties of reinforced wave transparent composites was studied. FTIR spectra revealed the Si-O peak while –OH, C = O, C-H, and C-N-C peaks were also found. Their amorphous nature was revealed by XRD while the SEM images showed the composite morphology along with fiber–matrix adhesion. Increased proportion of SiO_2 (f)_ (SF = x at 30 to 90 w/w%) resulted in excellent transmission, low εr from 3.85 to 3.12, and low δ from 0.07 to 0.036. With increased SiO_2 (f)_ (at x = 30 to 80), the UTS, UCS, and bending strength was increased, however, with more than optimum addition of SiO_2 (f)_ (x = 90) in the composite, the mechanical properties were reduced by 4.3% (UTS), 1% (UCS), and 1.7% (bend), respectively. SFE composite thermal stability was improved with the addition of SiO_2 (f)_, as 24% weight loss was seen (x = 90) whereas 69% weight was lost (x = 30) while low moisture absorption was found from 1.6 to 1.8%. In conclusion, this silica fabric can be a useful, promising, and cost-effective reinforcement for fabricating a reinforced composite, exhibiting excellent dielectric properties, good mechanical and thermal stability, and low moisture absorption.

## Figures and Tables

**Table 1:** Thermoset resin for dielectric applications.

Parameter	Cyanate Ester Resin	DGEBA Resin	Polyester Resin	Bismaleimide Resin
Density (g/cc)	1.17	1.30	1.29	1.30
εr	2.8–3.2	3.7–4.1	2.8–4.0	3.1–3.5
δ	0.002–0.008	0.018–0.020	0.006–0.026	0.005–0.020
Flexural strength (MPa)	80	97	85	150
Cost ratio (approx)	1:1	1:0.2	1:0.15	1:0.9

**Table 2:** Silica fibers for dielectric applications.

Parameter	Quartz-Glass	High-Silica Glass	D-Glass	E-Glass
SiO_2_%	99.99	95–98	72–75	52–57
Density	2.20	2.15	2.6	2.54
UTS (GPa)	1.70	1.8	2.4	3.75
εr	3.78	4.1	4.0	6.13
δ	0.0002	0.020	0.0025	0.0038
Cost ratio (approx)	1:3	1:1	1:2	1:1.2

**Table 3:** Experimental conditions—SFE composites fabrication.

Fabric Drying	Resin (R: H)	Degassing (5 min)	Soaking	Hot Pressing	Curing	Post Curing
120 °C	1.6:1	5 × 10^−3^ mbar	20 min	100 bar	140@4 h	130@3 h

**Table 4:** Identification scheme for SFE composites.

Identity	SF3E7	SF4E6	SF5E5	SF6E4	SF7E3	SF8E2	SF9E1
Silica%	30	40	50	60	70	80	90
Epoxy%	70	60	50	40	30	20	10

**Table 5:** Comparison of the properties of glass fiber epoxy composites (low dielectric application).

Parameter	D-GFs	E-GFs	Quartz-GFs	High-Silica-GFs
SiO_2_% (wt.)	≥72	≥54	≥99.9	≥96.5
Density (g/cc)	≤1.78	≤1.74	≤1.73	≤1.76
XRD (2ϴ) range	20°~22°	20°~22°	20°~22°	20°~22°
ε	2.8–3.4	5.4–6	2.95~3.3	3.17~3.82
δ	0.001~0.01	0.06~0.08	0.001~0.005	0.07~0.036
Compression (Mpa)	461.63	725.61	442.2	382.8~405.1
Tensile Strength (Mpa)	144.35	198.49	103.91	132.3~155.7
Flexural Strength(Mpa)	141.6	189.6	113.6	277.7 to 311.9
% Weight loss (TGA)	25.2	21.3	18.9	26.5
Moisture absorption%	1.3	2.3	1.1	1.1~1.3
Cost Ratio	Highest	Medium	High	Low
References	[33,34,35,36,37,38,39,40,41,42,45,46,47,48]			This work

## Data Availability

All data is contained within the article.
